# Tamoxifen-resistant breast cancer cells are resistant to DNA-damaging chemotherapy because of upregulated BARD1 and BRCA1

**DOI:** 10.1038/s41467-018-03951-0

**Published:** 2018-04-23

**Authors:** Yinghua Zhu, Yujie Liu, Chao Zhang, Junjun Chu, Yanqing Wu, Yudong Li, Jieqiong Liu, Qian Li, Shunying Li, Qianfeng Shi, Liang Jin, Jianli Zhao, Dong Yin, Sol Efroni, Fengxi Su, Herui Yao, Erwei Song, Qiang Liu

**Affiliations:** 1 Guangdong Provincial Key Laboratory of Malignant Tumor Epigenetics and Gene Regulation, Sun Yat-Sen Memorial Hospital, Sun Yat-Sen University, 510120 Guangzhou, China; 2 Breast Tumor Center, Sun Yat-Sen Memorial Hospital, Sun Yat-Sen University, 510120 Guangzhou, China; 3 Medical Research Center, Sun Yat-Sen Memorial Hospital, Sun Yat-Sen University, 510120 Guangzhou, China; 4 Faculty of Life Sciences, Bar-Ilan University, 52900 Ramat Gan, Israel

## Abstract

Tamoxifen resistance is accountable for relapse in many ER-positive breast cancer patients. Most of these recurrent patients receive chemotherapy, but their chemosensitivity is unknown. Here, we report that tamoxifen-resistant breast cancer cells express significantly more BARD1 and BRCA1, leading to resistance to DNA-damaging chemotherapy including cisplatin and adriamycin, but not to paclitaxel. Silencing BARD1 or BRCA1 expression or inhibition of BRCA1 phosphorylation by Dinaciclib restores the sensitivity to cisplatin in tamoxifen-resistant cells. Furthermore, we show that activated PI3K/AKT pathway is responsible for the upregulation of BARD1 and BRCA1. PI3K inhibitors decrease the expression of BARD1 and BRCA1 in tamoxifen-resistant cells and re-sensitize them to cisplatin both in vitro and in vivo. Higher BARD1 and BRCA1 expression is associated with worse prognosis of early breast cancer patients, especially the ones that received radiotherapy, indicating the potential use of PI3K inhibitors to reverse chemoresistance and radioresistance in ER-positive breast cancer patients.

## Abstract (web summary)

Most breast cancer patients are estrogen receptor positive and thus benefit from treatments that inhibit estrogen production; however, one third of tamoxifen-treated patients develops resistance and relapse. Here the authors show that tamoxifen resistant cells are resistant to chemotherapy because of BARD1 and BRCA1 upregulation.

## Introduction

Breast cancer (BC) is one of the leading causes of cancer morbidity and mortality in women worldwide. About 70% of breast cancer patients are estrogen receptor (ER) positive and thus benefit from endocrine treatments including ER antagonist tamoxifen and aromatase inhibitors that inhibit estrogen production. Tamoxifen, the most widely used adjuvant endocrine therapy, has been shown to substantially reduce the recurrence rate by ~40% and the mortality rate by ~30% in ER-positive breast cancer patients^1^. However, even with 5-years use of tamoxifen, one-third of these patients still relapse within 15 years^1^. These endocrine-resistant patients may represent up to one-quarter of all breast cancer patients^2^, presenting a huge clinical challenge.

Tremendous effort has been made to investigate the mechanism of tamoxifen resistance. It has been reported that tamoxifen resistance is often caused by activation of alternative growth pathway to overcome the growth suppression brought by tamoxifen. These pathways include PI3K/Akt/mTOR pathway^3–5^, cyclin D1/CDK4/6 pathway^6–10^, fibroblast growth factor receptor pathway^11,12^, IGF-1 pathway^13^, etc. Inhibitors of mTOR and CDK4/6 have already been approved by FDA to be used to treat endocrine-resistant breast cancers. In addition to the capacity to overcome tamoxifen-induced growth suppression, tamoxifen-resistant cells (TamR cells) also gain some aggressive biological features, such as an epithelial-to-mesenchymal transition (EMT) phenotype^14–16^ and some stem-cell-like properties^17^. For example, highly expressed CD44^18^ and SOX2^19^ were reported to promote tamoxifen resistance by increasing the proportion of stem/progenitor cells, which may also affect the response to other treatments such as chemotherapy.

In the treatment of recurrent ER-positive and Her2-negative breast cancer patients, several guidelines including NCCN and ABC3^20^ recommend endocrine therapy with or without targeted therapy to be the preferred treatment option. Chemotherapy is only recommended upfront for the patients with visceral crisis or concern/proof of endocrine resistance. Nevertheless, most of these patients are incurable and inevitably develop resistance to various kinds of endocrine treatments, and eventually need chemotherapy to control the disease. On the other hand, for recurrent ER-positive and Her2-positive breast cancers, anti-Her2 therapy plus chemotherapy is generally the favorable treatment. However, it has never been reported whether prior endocrine resistance could affect the response to chemotherapy in these patients. In this study, we show that tamoxifen-resistant breast cancer cells are resistant to DNA-damaging chemotherapy because of upregulated BARD1 and BRCA1, which is caused by activated PI3K/AKT pathway. Our results indicate an important role of BARD1/BRCA1 in chemoresistance of ER-positive breast cancer patients.

## Results

### Upregulated BARD1/BRCA1 in TamR breast cancer cells

**Fig. 1:**
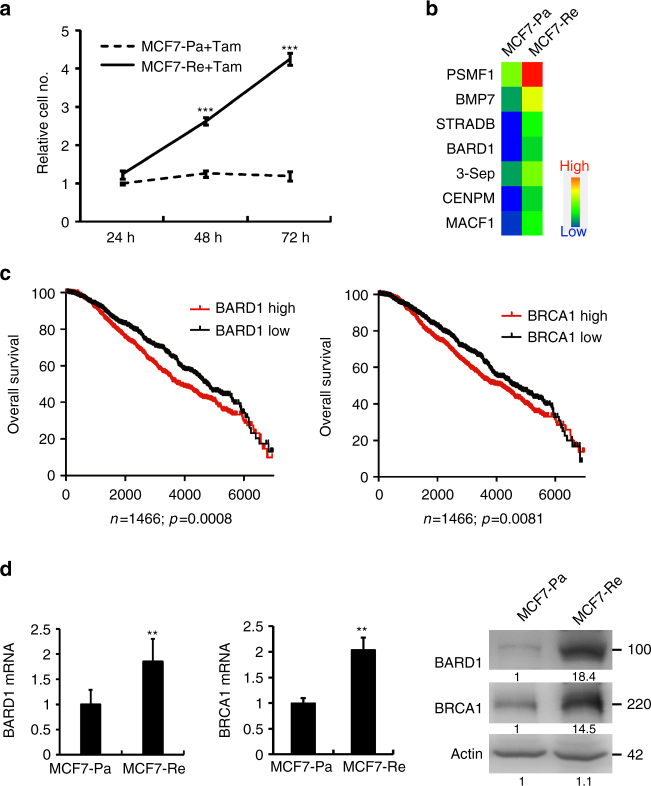
BARD1/BRCA1 is upregulated in tamoxifen-resistant breast cancer cells. **a** The growth curve of MCF7-Pa and MCF7-Re cells under tamoxifen treatment. **b** mRNA expression profile analysis shows upregulated proliferation-related genes in MCF7-Re cells. **c** Kaplan–Meier survival curves of breast cancer patients with low (black) and high (red) BARD1 or BRCA1 expression based on Curtis data set. **d** The mRNA and protein expression of BARD1 and BRCA1 in MCF7-Pa and MCF7-Re breast cancer cells. In **a**, **d**, data show means ± s.d. (*n* = 3) **p < 0.01; ***p < 0.001 compared with MCF7-Pa by Student’s *t* test

Tamoxifen-resistant breast cancer cell lines were established from MCF7 and T47D as reported before^21^ (Fig. 1a and Supplementary Fig. 1a). In order to identify genes that may be responsible for tamoxifen resistance, we used mRNA array to analyze the mRNAs that were differentially expressed between MCF7 tamoxifen-resistant cell line (MCF7-Re) and MCF7 parent cell line (MCF7-Pa). Since ER-independent proliferation is an essential feature of tamoxifen-resistant cells, we analyzed the proliferation-related genes by GO analysis and found that seven genes were upregulated more than twofolds in MCF7-Re compared to MCF7-Pa (Fig. 1b).

To examine the clinical significance of these upregulated genes, their prognostic values were analyzed using publicly available microarray data resources including oncomine and GEO data sets. We found that higher expression of BARD1 was associated with decreased overall survival (OS) in ER-positive breast cancer patients from publicly available Curtis breast cancer data set^22^ published on oncomine platform (Fig. 1c) and in the meta-analysis of breast cancer patients from 27 data sets, many of which do not provide the details of ER positivity (Supplementary Fig. 2a). qPCR and western blot confirmed that the mRNA and protein levels of BARD1 in MCF7-Re cells were significantly higher than those in MCF-Pa cells (Fig. 1d).

BARD1, the BRCA1-associated ring domain 1 protein, often works with BRCA1 by forming a heterodimer to regulate DNA damage repair, cell growth, and ubiquitin ligase activity^23–27^. Thus, we also examined the expression of BRCA1 and found that both mRNA and protein expression of BRCA1 in MCF7-Re were significantly higher than those in MCF-Pa (Fig. 1d). Similar upregulation of BARD1 and BRCA1 were also seen in T47D-Re compared with T47D-Pa cells (Supplementary Fig. 1b–d). Moreover, higher expression of BRCA1 was associated with significant shorter OS in ER-positive breast cancer patients from Curtis breast published on oncomine platform (Fig. 1c) and the meta-analysis of breast cancer patients from 27 data sets in GEO database (Supplementary Fig. 2b).

### Reduced BARD1/BRCA1 did not affect tamoxifen sensitivity

**Fig. 2:**
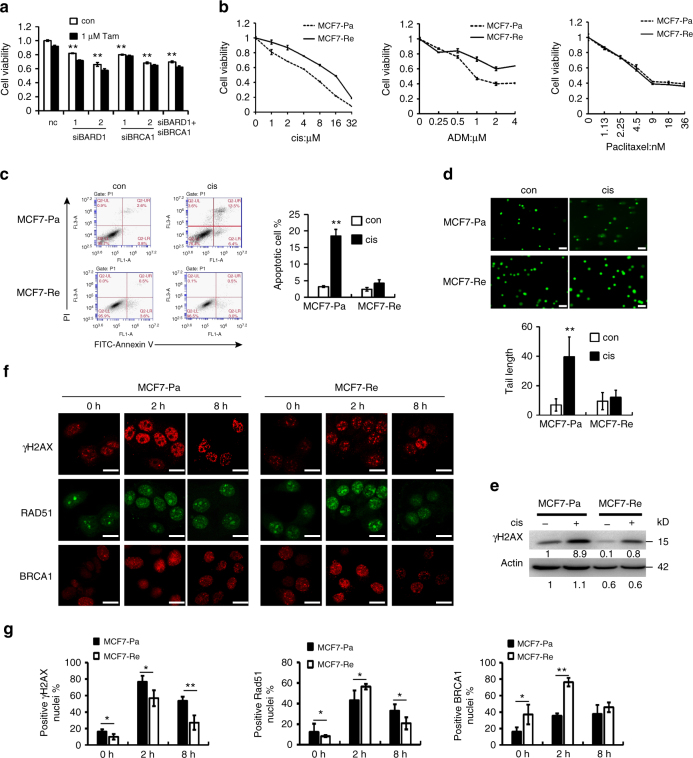
Tamoxifen-resistant cells are resistant to DNA-damaging chemotherapy. **a** Cell viability assay showing the effect of tamoxifen on MCF7-Re cells when transfected with negative control siRNA (nc), BARD1, and/or BRCA1 siRNAs. **b** MCF7-Pa and MCF7-Re cells were treated with different concentrations of cisplatin (cis), adriamycin (ADM), or paclitaxel, and the proportions of viable cells were examined by cell viability assay. **c** Apoptosis assay by AnnexinV-FITC/PI staining showing the percentages of apoptotic cells when MCF7-Pa and MCF7-Re cells were treated with cisplatin. **d** Comet assay showing the DNA damage when MCF7-Pa and MCF7-Re cells were treated with cisplatin. Scale bar indicated 100 μm. **e** Western blot showing the expression of γH2AX when MCF7-Pa and MCF7-Re cells were treated with cisplatin. **f** Representative immunofluorescence staining of γH2AX, Rad51, and BRCA1 in MCF7-Pa and MCF7-Re cells treated with cisplatin at 0 h, 2 h, 8 h. Scale bar indicated 25 μm. **g** Percentages of positive nuclei (10 foci or more per nucleus being considered positive) from three fields per sample are indicated. In **a**–**d**, **g**, data show means ± s.d. (*n* = 3) *p < 0.05; **p < 0.01; compared with control or indicated lines by Student’s *t* test

To determine whether increased BARD1/BRCA1 expression contributes to tamoxifen resistance, BARD1 or BRCA1 were knocked down with siRNAs (Supplementary Fig. 1e, f), and the cell growth and tamoxifen sensitivity of MCF7-Re cells were evaluated using MTT assay. We found that reduced BARD1 or BRCA1 slowed the growth of MCF7-Re cells by 20–30%, but had no effect on their tamoxifen sensitivity (Fig. 2a). Knocking down of BARD1 and BRCA1 together also showed similar effect, suggesting that increased BARD1 and BRCA1 may not play a major role in tamoxifen resistance.

### TamR cells were resistant to DNA-damaging chemotherapy

Both *BRCA1* and *BARD1* are known to be tumor suppressor genes, whose mutations are associated with an increased risk of breast cancer. The mutation of BRCA1 or BARD1 results in defective DNA damage repair, which also lead to increased sensitivity to platinum-based chemotherapy. Since the expression of BARD1 and BRCA1 was significantly increased in MCF7-Re and T47D-Re cells, we examined whether these tamoxifen-resistant cells would also be resistant to chemotherapy.

MCF7-Re or MCF7-Pa cells were treated with increasing concentrations of three major chemotherapeutic drugs used in breast cancer, including cisplatin, adriamycin, and paclitaxel (Fig. 2b). MCF7-Re cells (IC_50_ = 13 μM) were markedly more resistant to cisplatin than MCF7-Pa cells (IC_50_ = 4 μM, *p* < 0.001, compared with MCF7-Re cells by Student's *t *test). Similar results were obtained when MCF7-Re or MCF7-Pa cells were treated with adriamycin, with IC_50_ being 4 μM for MCF7-Re and 0.7 μM for MCF7-Pa (*p* < 0.001, compared with MCF7-Re cells by Student's *t* test). Nevertheless, there was no difference in the sensitivity to paclitaxel between MCF7-Re and MCF7-Pa cells. Apoptosis assay also showed that cisplatin induced less apoptotic cells in MCF7-Re cells than in MCF7-Pa cells (Fig. 2c).

It is known that both cisplatin and adriamycin can induce DNA damage, while paclitaxel cannot. To determine whether the resistance to cisplatin and adriamycin in MCF7-Re cells was mediated by enhanced DNA damage repair, we measured the expression of γH2AX and did comet assay to examine the DNA damage in the cells. Cisplatin significantly induced the expression of γH2AX, a marker of DNA damage, in both MCF7-Pa and MCF7-Re cells. However, γH2AX was noticeably lower in MCF7-Re cells than that in MCF7-Pa cells, indicating less DNA damage was caused in MCF7-Re cells (Fig. 2e). Comet assay, a sensitive technique to detect DNA damage at the level of an individual cell, also showed that cisplatin induced significant tail (damaged DNA) in MCF7-Pa cells, but not in MCF7-Re cells (Fig. 2d). Furthermore, T47D-Re cells were also more resistant to cisplatin and adriamycin than T47D-Pa cells, but not to paclitaxel (Supplementary Fig. 3a, b). These results suggest that DNA-damaging chemotherapeutic drugs induced significantly less DNA damage in tamoxifen-resistant breast cancer cells.

To further study whether the above phenomenon is caused by less induction or faster repair of DNA damage, we used immunofluorescence staining to measure the numbers of γH2AX, RAD51, and BRCA1 foci in MCF7-Pa and MCF7-Re cells after cisplatin treatment at 0 , 2, and 8 h. We found that the numbers of γH2AX and RAD51 foci were lower, while the number of BRCA1 foci was higher in MCF7-Re than MCF7-Pa cells before cisplatin treatment (0 h), consistent with higher BRCA1 expression and lower DNA damage in MCF7-Re cells. Cisplatin treatment for 2 h significantly increased the numbers of γH2AX, RAD51, and BRCA1 foci in both MCF7-Pa and MCF7-Re cells. But the numbers of γH2AX, RAD51, and BRCA1 foci in MCF7-Re cells significantly diminished after cisplatin treatment at 8 h, while these foci in MCF7-Pa cells remained at high level or decreased much slower (Fig. 2f, g). These results indicate that the induction of DNA damage repair foci is similar between MCF7-Pa and MCF7-Re cells, whereas MCF7-Re cells have higher efficiency to repair damaged DNA than MCF7-Pa cells.

### Reduced BARD1/BRCA1 re-sensitized TamR cells to chemotherapy

**Fig. 3:**
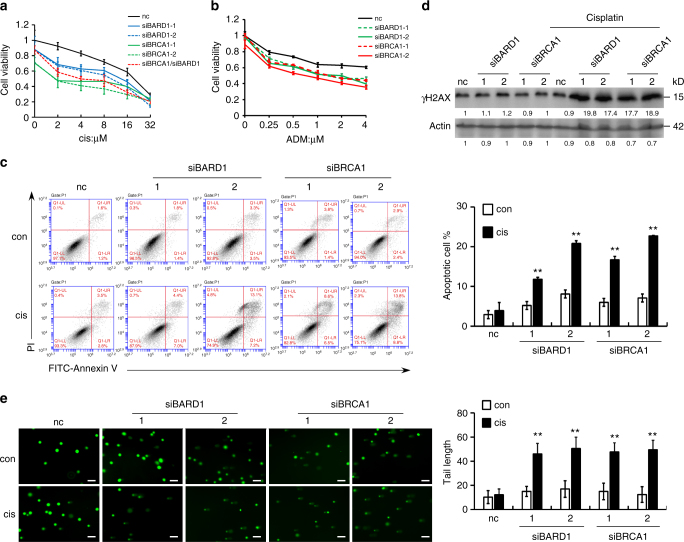
Reduced BARD1 or BRCA1 expression sensitizes tamoxifen-resistant cells to cisplatin. MCF7-Re cells were transfected with negative control siRNA (nc), BARD1, and/or BRCA1 siRNAs. **a**,** b** Cell viability assay showing the sensitivity to cisplatin (**a**) and ADM (**b**). **c** Apoptosis assay by AnnexinV-FITC/PI staining showing the effect of cisplatin on the percentages of apoptotic cells. **d** The expression of γH2AX induced by cisplatin. **e** Comet assay showing the DNA damage induced by cisplatin. Scale bar indicated 100 μm. In **a**–**c**, **e**, data show means ± s.d. (*n* = 3) **p < 0.01 compared with control by Student’s *t* test

To determine whether increased expression of BARD1 and BRCA1 is responsible for the faster repair of DNA damage and resistance to DNA-damaging chemotherapy in tamoxifen-resistant cells, we knocked down the expression of BARD1 or BRCA1 using siRNAs in MCF7-Re cells and measured their sensitivity to cisplatin and adriamycin. As expected, silencing of either BARD1 or BRCA1 significantly re-sensitize MCF7-Re cells to cisplatin (IC_50_ = 8.2/6.2 μM for BARD1-si1/si2 and IC_50_ = 2.5/1.7 μM for BRCA1-si1/si2), compared with control siRNA group (IC_50_ = 15.5 μM, *p* < 0.001, compared with nc by Student's *t* test). Silencing of both BARD1 and BRCA1 did not have additive effect, indicating that these two proteins work through the same pathway (Fig. 3a). Similar effects were obtained with adriamycin (Fig. 3b). In addition, flow cytometry also showed that cisplatin induced significantly more apoptosis in MCF7-Re cells upon the transfection of siRNAs targeting BARD1 or BRCA1 (Fig. 3c). Assays of DNA damage, γH2AX and comet assay, demonstrated that silencing of BARD1 or BRCA1 rendered MCF7-Re cells more vulnerable to cisplatin-induced DNA damage (Fig. 3d, e). Reversal of cisplatin resistance by siRNAs targeting BARD1 and/or BRCA1 was also seen in T47D-Re cells (Supplementary Fig. 3c). Together, these results indicate that increased BARD1 and BRCA1 expression leads to enhanced DNA damage repair in tamoxifen-resistant cells and results in their resistance to DNA-damaging chemotherapy.

To rule out the off-target effects of siRNAs, we constructed expression vectors of BARD1 and BRCA1 that do not have 3′-UTR and thus are resistant to siRNAs. We found that the expression of these vectors restored BARD1 or BRCA1 expression in siRNAs-treated MCF7-Re cells (Supplementary Fig. 4a), and also made the cells resistant to cisplatin again (Supplementary Fig. 4b–d), suggesting the re-sensitizing effect of these siRNAs is not off-target effect.

Since double-strand breaks induced by adriamycin can also be repaired by non-homologous end joining pathway, we measured the mRNA expression of non-homologous end joining (NHEJ) pathway-associated genes including *LIG4*, *XRCC4*, *XRCC5*, *XRCC6*, and *XRCC7* (Supplementary Fig. 5a). We found that LIG4 and XRCC4 expressions were markedly lower, while XRCC6 expression was higher in MCF7-Re cells than in MCF7-Pa cells. The expression of XRCC5 and XRCC7 was similar in MCF7-Pa and MCF7-Re cells. To further study the possible role of NHEJ in chemoresistance of MCF7-Re cells, we used two siRNAs to knockdown LIG4 (Supplementary Fig. 5b, c), the key DNA ligase in NHEJ pathway that joins double-strand breaks, and found that decreased LIG4 did not change the sensitivity to adriamycin in MCF7-Re cells. Furthermore, SCR7, a DNA ligase IV inhibitor, also had no impact on the sensitivity to adriamycin in MCF7-Re cells (Supplementary Fig. 5d, e). These results suggest that NHEJ pathway may not play an important role in chemosensitivity of tamoxifen-resistant breast cancer cells.

### PI3K or CDK1/12 inhibition led to BRCA1/BARD1 downregulation

To overcome the chemoresistance of tamoxifen-resistant breast cancer cells, it is necessary to identify the mechanism of BARD1 and BRCA1 upregulation that are targetable. It was reported that PI3K/Akt pathway controls DNA damage repair^28,29^. PI3K inhibition led to decreased BRCA1/2 expression and induced homologous recombination deficiency, which sensitized BRCA-proficient triple negative breast cancer to PARP inhibition^30^. It is also known that activating mutation of PI3K pathway is one of the most common genetic events in breast cancer, most notably in endocrine-resistant ER-positive breast cancer^31^.

**Fig. 4:**
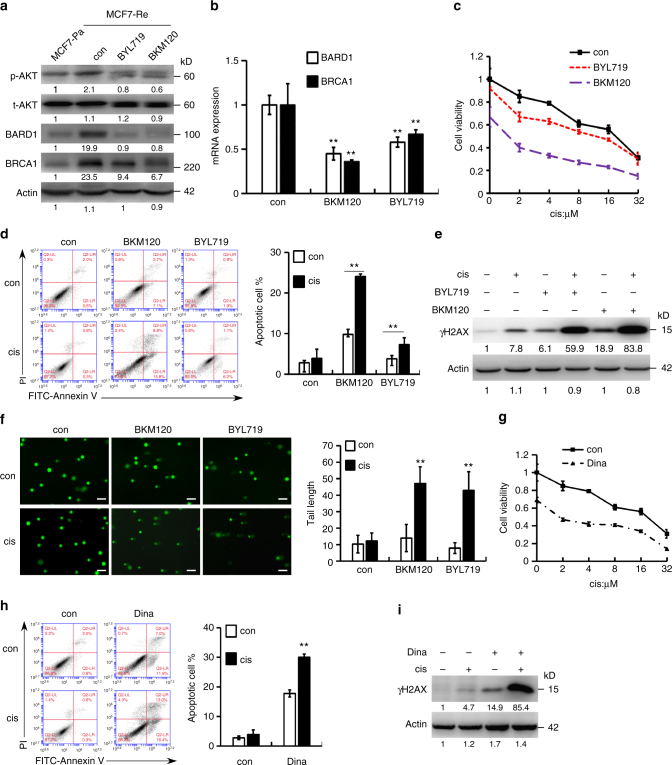
PI3K inhibition decreases BRCA1/BARD1 expression and sensitizes tamoxifen-resistant cells to cisplatin. **a**, **b** The protein (**a**) and mRNA (**b**) expression of BARD1/BRCA1 in MCF7-Re cells treated with BKM120 or BYL719. **c**–**f** MCF7-Re cells were treated with control, BKM120, or BYL719 and then treated with cisplatin. The sensitivity to increasing concentrations of cisplatin (**c**), apoptotic cells (**d**), γH2AX expression (**e**), and DNA damage shown by comet assay (**f**) were measured. Scale bar indicated 100 μm. **g**–**i** MCF7-Re cells were treated with Dinaciclib and cisplatin. The sensitivity to increasing concentrations of cisplain (**g**), apoptotic cells (**h**), and γH2AX expression (**i**) were measured. In **b**–**d**, **f**–**h**, data show means ± s.d. (*n* = 3) **p < 0.01 compared with control by Student’s *t* test

Indeed, we found that the expression of phosphorylated Akt was significantly higher in MCF7-Re cells than MCF7-Pa cells, indicating a hyper-activated PI3K pathway in MCF7-Re cells (Fig. 4a). Moreover, BKM120 (a pan-PI3K inhibitor) and BYL719 (a selective PI3Kα inhibitor) significantly inhibited the phosphorylation of Akt, as well as the mRNA and protein expression of BARD1 and BRCA1 (Fig. 4a, b), suggesting that activated PI3K/Akt pathway is responsible for the upregulated BARD1 and BRCA1 in tamoxifen-resistant cells. Furthermore, the two PI3K inhibitors, BKM120 and BYL719, also markedly sensitized MCF7-Re cells to cisplatin treatment (Fig. 4c). Cisplatin with PI3K inhibition induced obviously more apoptosis (Fig. 4d) and more DNA damage (Fig. 4e, f) than cisplatin alone in MCF7-Re cells, suggesting that PI3K inhibitors can inhibit DNA damage repair and re-sensitize tamoxifen-resistant cells to DNA-damaging chemotherapy.

Additionally, we found that inhibition of PI3K pathway by BKM120 and BYL719 partially restored the sensitivity to tamoxifen (Supplementary Fig. 6a), indicating the activation of PI3K pathway also contributes to tamoxifen resistance of MCF7-Re cells. To further explore the impact of cell cycle dysregulation in BARD1/BRCA1 expression, we analyzed the cell cycle distribution of MCF7-Pa cells and MCF7-Re cells. We found that MCF7-Re cells had significantly higher proportion of G2/M cells than MCF7-Pa cells, which was suppressed to the level of MCF7-Pa cells by BKM120 and BYL719 (Supplementary Fig. 6b). Using thymidine (Th) and/or nocodazole (NOC) treatment to synchronize MCF7 cells at G1, S, and G2/M phases (Supplementary Fig. 6c), we found that BRCA1 and BARD1 were expressed throughout the cell cycle, but cells in G1 phase have lower expression of these proteins than cells in G2/M and S phase (Supplementary Fig. 6d). These results are consistent with previous reports^32^ and explain why BARD1 and BRCA1 are upregulated in MCF7-Re cells.

In addition to PI3K/Akt pathway, CDK1 activity was also reported to be critical for BRCA1 phosphorylation, an event essential for efficient BRCA1 focus formation and DNA damage repair^33^. Inhibition of CDK1 sensitized BRCA1-proficient cancer to PARP inhibitor^34^. Thus, we examined whether CDK1 inhibition can re-sensitize tamoxifen-resistant cells to cisplatin. Dinaciclib, an inhibitor of CDKs including CDK1, efficiently reduced BRCA1 phosphorylation and expression as reported (Supplementary Fig. 7a), induced more DNA damage and apoptosis with cisplatin, and significantly increased the sensitivity to cisplatin in MCF7-Re cells (IC_50_ = 13 μM for control and IC_50_ = 1.8 μM for Dinaciclib) (Fig. 4g–i).

Dinaciclib was recently shown to be also a CDK12 inhibitor that can reduce BRCA1 transcription^35^. To dissect the role of CDK1 and CDK12 in dinaciclib’s reversal of chemoresistance, we examined the effects of both siCDK1 and siCDK12 in the induction of γH2AX, cell apoptosis, response to cisplatin, and BRCA1 expression. We found that CDK1 knockdown did not change the expression of BRCA1, but decreased the phosphorylation of BRCA1 (Supplementary Fig. 7b, d). Nevertheless, silencing of CDK12 inhibited BRCA1 mRNA and protein expression (Supplementary Fig. 7c, d). In γH2AX and apoptosis assay, both siCDK1 and siCDK12 led to the re-sensitization to cisplatin in MCF7-Re cells (Supplementary Fig. 7e, f). Thus, inhibition of both CDK1 and CDK12 by Dinaciclib seems to be important in the reversal of chemoresistance in breast cancer cells.

Similar to that in MCF7-Re cells, the two PI3K inhibitors, BKM120 and BYL719, significantly inhibited Akt phosphorylation and the expression of BARD1 and BRCA1 and restored the sensitivity to cisplatin in T47D-Re cells, another tamoxifen-resistant cell line (Supplementary Fig. 8a–d). Dinaciclib also re-sensitized T47D-Re cells to cisplatin treatment (Supplementary Fig. 8e). Together, these results indicate that the inhibition of PI3K or CDK1/12 can reverse the chemoresistance in tamoxifen-resistant breast cancer cells.

### PI3K inhibitor re-sensitized TamR xenografts to cisplatin

**Fig. 5:**
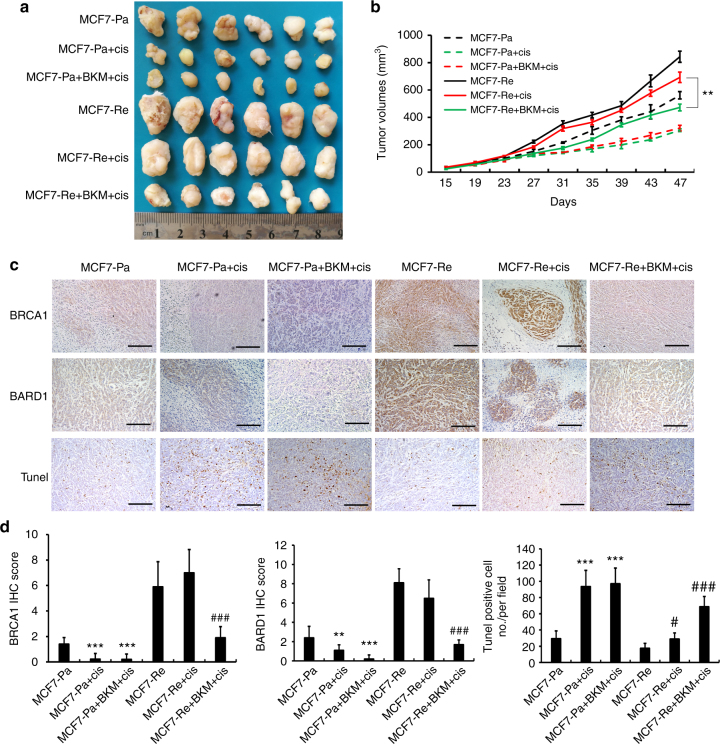
PI3K inhibitor restores the sensitivity to cisplatin in xenografts of tamoxifen-resistant tumor in vivo. MCF7-Pa and MCF7-Re cells were inoculated into the mammary fat pads of Balb/c nude mice. When tumors became palpable, tumors were treated with control, cisplatin, or cisplatin with BKM120. The picture (**a**), tumor volume (**b**), and immunohistochemical staining of BARD1/BRCA1 and Tunel staining (**c**) of xenografts derived from MCF7-Pa and MCF7-Re cells with indicated treatment. **d** IHC scores of BRCA1 and BARD1 and Tunel-positive cell number counts in indicated xenografts. In **b**, **d**, data show means ± s.d. (*n* = 6) **p < 0.01 by Student’s *t* test. Scale bar indicated 400 µm

To validate these results in vivo, we inoculated MCF7-Pa and MCF7-Re cells into the mammary fat pads of Balb/c nude mice. Tumors from MCF7-Pa cells grew slower than tumors from MCF7-Re cells. By 47 days, the volume of MCF7-Re tumors reached 843 mm^3^, and the MCF7-Pa tumors were 475 mm^3^ (Fig. 5a, b). When tumors were palpable, they were treated with control solution, cisplatin alone, or cisplatin with BKM120 for 3 weeks. Cisplatin treatment significantly shrank the MCF7-Pa tumors, while had no obvious effect on the MCF7-Re tumors, indicating the chemoresistance of the latter. Furthermore, the treatment of BKM120 and cisplatin significantly decreased the size of MCF7-Re tumors compared with the treatment of cisplatin alone, suggesting that PI3K inhibitor restores the sensitivity to cisplatin in MCF7-Re tumors.

Immunohistochemical (IHC) staining showed that the expression of BRCA1 and BARD1 was markedly higher in MCF7-Re tumors than in MCF7-Pa tumors, and was decreased with BKM120 treatment. Tunel staining also demonstrated that apoptotic cells were significantly more in MCF7-Pa tumors after cisplatin treatment, whereas apoptotic cells only increased in MCF7-Re tumor with treatment of BKM120 and cisplatin, but not with cisplatin treatment alone (Fig. 5c, d).

### High BARD1/BRCA1 related to BC metastasis and poor prognosis

**Fig. 6:**
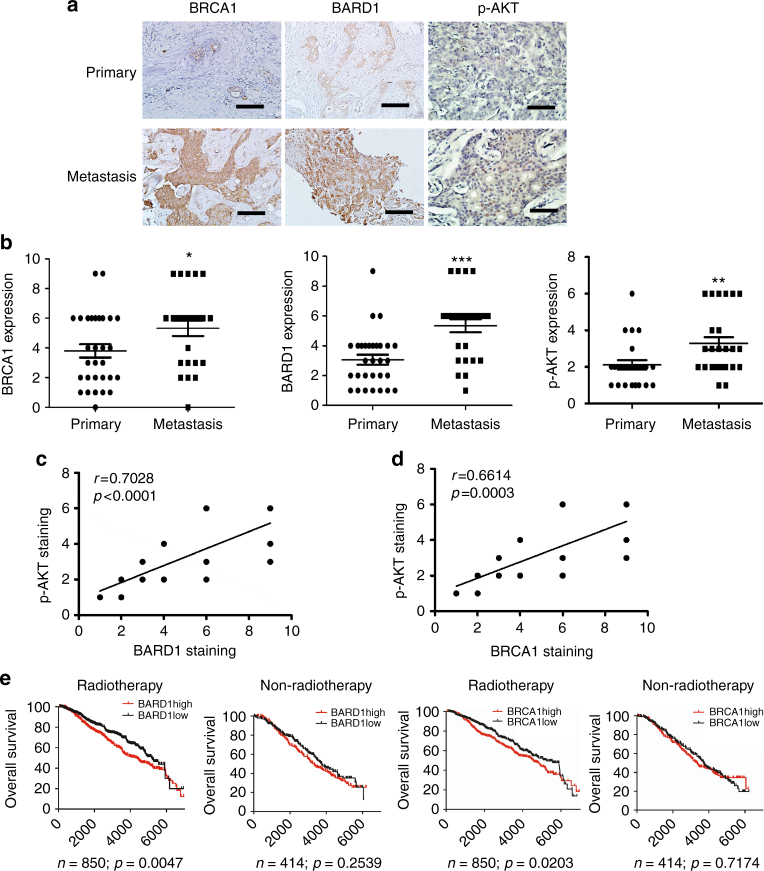
The expression of BARD1 and BRCA1 are increased in metastatic breast cancer and associated with poor prognosis in ER+ breast cancer patients received radiotherapy. **a**, **b** Representative pictures (**a**) and quantification (**b**) of IHC staining of BARD1, BRCA1, and p-AKT in paired primary and metastatic breast cancer tissues. **c**, **d** Correlation analysis of p-AKT expression with BARD1 (**c**) or BRCA1 (**d**) expression in primary and metastatic breast cancer samples. **e** Overall survival of breast cancer patients in Curtis data set with or without radiotherapy whose tumors had high or low BARD1/BRCA1 expression. In **b**, data show means ± s.d. (*n* = 27) *p < 0.05; **p < 0.01; ***p < 0.001 compared with that in primary breast cancer by Student’s *t* test. Scale bar indicated 400 µm

To further confirm the clinical relevance of the above findings, we analyzed the expression of BARD1 and BRCA1 in 27 paired primary and relapsed tumor samples from recurrent breast cancer patients who underwent adjuvant tamoxifen treatment and had their recurrent tumors biopsied or resected. IHC staining showed that the expression of BARD1 and BRCA1 was significantly increased in the recurrent tamoxifen-resistant tumors than that in primary tumors (Fig. 6a, b), in agreement with the finding of upregulated BARD1 and BRCA1 in tamoxifen-resistant breast cancer cell lines.

We then stained both the primary and metastatic breast cancer samples with anti-phosphorylated AKT (p-AKT) antibody to determine their PI3K pathway activation. We found that the expression of p-AKT was significantly higher in metastatic breast cancer samples than in primary tumors (Fig. 6a, b). Moreover, BARD1 and BRCA1 were positively correlated with p-AKT expression in primary and metastatic breast cancer samples (Fig. 6c, d), suggesting that BARD1/BRCA1 expression is associated with PI3K pathway activation.

Many breast cancer patients received chemotherapy including anthracycline, taxane, or both, but the details of chemotherapy were not included in oncomine data sets. Thus, it is difficult to determine the relationship between chemotherapy and the expression of BARD1 and BRCA1. Radiotherapy is another major adjuvant therapy used in early breast cancer and works mainly by inducing DNA damage. Interestingly, high BARD1 and BRCA1 expression was associated with poor prognosis in breast cancer patients receiving radiotherapy in Curtis data set, but had no significant prognostic effect in patients not receiving radiotherapy (Fig. 6e), further corroborating the notion that high BARD1 and BRCA1 expression results in resistance to DNA-damaging therapy including chemotherapy and radiotherapy.

## Discussion

In this study, we found that BARD1 and BRCA1 were highly expressed in tamoxifen-resistant breast cancer cells, which led to enhanced DNA damage repair and resistance to DNA-damaging chemotherapy. PI3K inhibitors reduced the expression of BARD1 and BRCA1, and restored the sensitivity to cisplatin in tamoxifen-resistant cells both in vitro and in vivo. More importantly, high expression of BARD1 and BRCA1 was associated with poor prognosis of ER+ breast cancer patients, especially in those received radiation therapy.

Most breast cancer patients are ER positive and receive adjuvant endocrine treatment including tamoxifen. Despite the high efficacy of tamoxifen, one-third of these patients still relapse after tamoxifen treatment. Chemotherapy is eventually needed for majority of these recurrent or metastatic breast cancer patients because resistance to various endocrine treatments is just a matter of time for advanced breast cancer. Previous studies indicate that tamoxifen resistance may enrich cancer cells with EMT or cancer stem/progenitor cells, which are also associated with chemoresistance. However, whether tamoxifen resistance changes the chemosensitivity of breast cancer has not been addressed. Our study, for the first time, showed that tamoxifen-resistant breast cancer cells are resistant to DNA-damaging chemotherapy, but not to paclitaxel. This suggests that microtubule-targeting drugs such as taxane may be superior to DNA-damaging drugs including anthracyclines and cisplatin/carboplatin when choosing chemotherapy for tamoxifen-resistant breast cancer patients.

BARD1, a protein that interacts with and stabilizes BRCA1, plays an important role in the rapid relocation of BRCA1 to DNA damage sites^25^. When the BRCA1/BARD1 heterodimer is transported to the damaged DNA site, it ubiquitinates RNA polymerase II and prevents the transcription of damaged DNA, thus restoring genetic stability^36^. Deleterious mutations of BRCA1 often cause disrupted BARD1/BRCA1 interaction, suggesting that the formation of a stable BARD1/BRCA1 complex may be an essential aspect of BRCA1 tumor suppression. Similar to BRCA1 mutations, BARD1 mutations also lead to increased risk of triple negative breast cancer and ovarian cancer. In contrast to the association between BRCA1/BARD1 mutation and increased risk of basal-like breast cancer, the analysis of multiple independent data sets showed that the expression of BARD1 and BRCA1 is associated with poor prognosis in ER-positive breast cancer patients, but not in ER-negative ones. These results indicate that BARD1 and BRCA1 have previously unappreciated prognostic value in luminal-type breast cancer, which may be related with its high prevalence of PI3K pathway activation.

PI3K/Akt/mTOR pathway is an intracellular signaling pathway that governs several key cellular processes, including proliferation, apoptosis, angiogenesis, and survival. Mutations of PI3K pathway are the most common genetic alterations in ER-positive early breast cancer^22^, as well as in recurrent or metastatic ones. These mutations often lead to the hyperactivation of PI3K pathway and play an important role in the resistance to endocrine therapy. Thus, PI3K/Akt/mTOR pathway is a hot target to overcome endocrine resistance, with mTOR inhibitor everolimus already approved by FDA and several PI3K inhibitors currently in clinical trials. It was recently reported that PI3K regulated BRCA1/2 expression via MAPK/ERK signaling and PI3K inhibition resulted in downregulated BRCA1/2. We also found that PI3K inhibitors significantly decreased the BARD1/BRCA1 expression in tamoxifen-resistant cells and re-sensitized them to chemotherapy. Since high BARD1/BRCA1 expression predicts poor prognosis in ER+ early breast cancer patients, it is possible that PI3K inhibitors not only help to overcome endocrine resistance, but may also reverse the resistance to DNA-damaging chemotherapy and radiotherapy in ER+ early breast cancer patients that have a 29–45% chance of PIK3CA mutation.

In summary, our study reveals the unacknowledged resistance to DNA-damaging chemotherapy in tamoxifen-resistant breast cancer cells, which can be overcome by PI3K inhibitors or choosing taxane-based chemotherapy. Moreover, this study provides the rationale to investigate whether PI3K inhibitors can be used in ER+ early breast cancer patients with activated PI3K pathway and upregulated BARD1/BRCA1 to increase their sensitivity to chemotherapy and radiotherapy, an approach that may increase the cure rate of those high-risk patients.

## Methods

### Cell culture

MCF7 and T47D breast cancer cells were obtained from the American Type Culture Collection (ATCC). MCF7 cell line was authenticated using short tandem repeat multi-amplification and tested to be mycoplasma negative. MCF7 cells were cultured in DMEM (Gibco, USA) supplemented with 10% FBS (Hyclone, USA) and 0.01 mg ml^−1^ human recombinant insulin (Sigma, USA). T47D cells were maintained in RPMI-1640 medium (Gibco, USA) supplemented with 10% FBS (Hyclone, USA).

### Establishment of tamoxifen-resistant cells

Tamoxifen-resistant MCF7 and T47D cells were established as previously reported^21,37,38^. Briefly, 2 × 10^5^ cells per well plated in 6-well plate and culture for 24 h (hrs). Then, the cells were washed with PBS, the culture medium was changed with phenol red free RPMI-1640 containing 5% charcoal-stripped steroid depleted FBS (Hyclone, USA). Aliquot of 0.1 μM 4-OH tamoxifen was added to the medium after 24 h culture. Finally, the concentration of 4-OH tamoxifen was gradually increased up to 1 μM. When the growth of the cells cannot be inhibited with 1 μM 4-OH tamoxifen, the tamoxifen-resistant cell line was established.

### Cell viability assays

MCF7 and T47D parental cells or tamoxifen-resistant cells were plated in 48-well plates in triplicates in charcoal-stripped medium (css) and treated with interest drugs (0–32 μM cisplatin, 0.5 μM BKM120, 1 μM BYL719, 0.5 μM dinaciclib) for 48 h. Then, the cell viability was detected using MTS regents (Promega, USA) according to the manufacturer’s recommendations.

### qPCR

Total RNA were extracted from breast cancer cells using TRIzol® Reagent (Life Technologies, USA), and RNA concentration and quality were determined by the absorbance of RNA at 260 and 280 nm. RNA was reverse-transcribed into cDNA using M-MLV Reverse Transcriptase (Life Technologies, USA). Real-time PCR was carried out using SYBR® Premix Ex TaqTM II (Takara, Japanese) according to the manufacturer’s recommendations and reactions were carried out in LightCycler480 system.

### Western blot analysis

Cells were lysed in RIPA lysis buffer (Beotime, China) supplemented with protease and phosphatase inhibitors (Life Technologies, USA). Protein samples were subjected to 8% SDS-PAGE and transferred to PVDF membranes (Bio-Rad, USA). Membranes were then blocked with 5% non-fat milk in 0.1% TBST buffer overnight at 4 °C. The membranes were subsequently incubated with BRAD1 (sc-74559, Santa Cruz, 1:1000), BRCA1 (OP92, Calbiochem, 1:500), p-BRCA1 (sc-24512, Santa Cruz, 1:500), CDK1 (9116, CST, 1:1000), CDK12 (11973, CST, 1:1000), Lig4 (ab26039, Abcam, 1:1000), AKT (4685, CST, 1:1000), P-AKT (2965, CST, 1:1000), H2AX/γH2AX antibody (2577/2599, CST, 1:1000). The protein–antibody complex was detected using HRP-conjugated secondary antibodies and enhanced chemiluminescence (Pierce). The band intensity was quantified using ImageJ software (NIH, Bethesda). Uncropped scans of western blots presented in the main figures are provided in Supplementary Figs 9–13.

### Small interfering RNA studies

Cells were plated in css medium without antibiotics at 2 × 10^4^ per well on a 24-well plate. Cells were then transfected with 50 nmol l^−1^ negative control small interfering RNA (siRNA), or 50 nmol l^−1^ BARD1 siRNA or BRCA1 siRNA (RiboBio, China) using Lipofectamine® RNAiMAX Transfection Reagent (Life Technologies, USA). Medium was changed after 6 h and every other day thereafter until the end of the experiment.

### Apoptosis assays

Cells were treated with 8 µM cisplatin or 0.1% DMSO as a control for 48 h. After harvest, cells were subsequently treated with AnnexinV-FITC/PI Apoptosis Kit (Multisciences, China) and analyzed by flow cytometry (BD Accuri™ C6).

### Cell cycle analysis

Cells were harvested and fixed in 70% ethanol for more than 24 h and stained with propidium iodide (PI). Cells were synchronized at the G1/S boundary by incubation with 5 mM thymidine (Th) for 12 h, followed by 12 h of incubation in growth medium prior to the addition of 5 mM Th (12 h). Then, cells were released from Th block in growth medium containing 100 nM nocodazole (NOC) (12–14 h). To release cells from NOC-induced mitotic arrest, the cells were washed and 10% FBS-containing medium was added for 18 h.

### DNA damage assay

Cells cultured in 24-well plate were treated with cisplatin or PI3K inhibitors (BKM120 or BYL719) for 24 h. DNA damage was then evaluated with the Comet assay kit (Trevigen, USA) following the instructions of the manufacture. Tail length was measured by using comet assay software project (CASP).

### Animal experiment

Female 4–6 weeks old NOD/SCID mice were used. All animal experiments were approved by Sun Yat-sen University laboratory animal care and use committee. 17β-Estrogen pellets (0.72 mg, 60-day release, innovative research of America) were implanted subcutaneously in 4–5-week female Balb/c nude mice 1 week before cell injection. Total of 1 × 10^7^ MCF7-Pa or MCF7-Re cells (suspended in 0.1 ml PBS) were inoculated in mammary fat pad. After tumors were palpable, tumor sizes were measured every 3 days and the tumor volume was calculated with the following formula: tumor volume (mm^3^) = length × width^2^ × 0.5. Once the tumors reached a mean size of ~100 mm^3^, the MCF-7 and MCF-7Re xenografts mice were randomly divided into three groups (six per group), respectively: (1) control group (PBS), (2) cisplatin group (ip, 4 mg kg^−1^, twice a week), (3) cisplatin+BKM120 group (ip, 4 mg kg^−1^ Cis, twice a week+oral gavage, 30 mg kg^−1^ BKM120, daily). Tumor volumes were monitored till the mice were killed. Mice were killed in a humane manner, and the tumors were collected for immunohistochemical staining.

### Immunohistochemistry

Paraffin-embedded samples were sectioned at 4-μm thickness. Antigen retrieval was performed by a pressure cooker for 30 min in 0.01 M citrate buffer (pH 6.0), followed by treatment with 3% hydrogen peroxide for 15 min. Specimens were blocked with normal goat serum for 30 min. Specimens were incubated with antibodies specific for BARD1 and BRCA1 overnight at 4 °C. Immunostaining was performed using DAB (Dako) according to the manufacturer’s instructions. The staining scores were determined based on both the intensity and proportion of indicated protein-positive cells in 10 random fields under a ×400 magnification. The proportion of positively stained tumor cells in sections was graded as follows: 0, no positive cells; 1, <10%; 2, 10–50%; and 3, >50%. The cells at each staining intensity were recorded on a scale of 0 (no staining), 1 (light brown), 2 (brown), and 3 (dark brown). The staining index (SI) was calculated as follows: SI = staining intensity × proportion of positively stained cells. Using this method, the expression of indicated protein was evaluated using SI and scored as 0, 1, 2, 3, 4, 6, or 9. Cells stained with Tunel were calculated per field of view, with at least 10 view fields per section were evaluated at ×400 magnification.

### Immunofluorescence

Cells seeded onto coated cover slips growth for 24 h, and then treated with cisplatin, harvested the cells at 0, 2, and 8 h. The cells were fixed with 4% paraformaldehyde for 15 min at room temperature. Then blocked with 10% goat normal serum+0.1% Triton X-100 in PBS for 30 min. Cells were incubated with primary antibodies overnight at 4 °C, followed by incubation with Alexa Fluor 594 goat anti-rabbit IgG (H+L) and Alexa Fluor 488 donkey anti-mouse IgG (H+L). Images were acquired using a confocal microscope.

### Statistical analysis

All in vitro statistical analyses were performed using Excel software. Results were expressed as means ± SD. Survival curve was performed using GraphPad Prism 6 software and results were expressed as log-rank test. *p* < 0.05 was considered significant.

### Data availability

The survival data were obtained from public oncomine portal (www.oncomine.org), GEO data sets, METABRIC, and E-MTAB. All other remaining data are available in the article and supplementary files.

## Electronic supplementary material

Supplementary Information
