# Supplementary material for: Tamoxifen-resistant breast cancer cells are resistant to DNA-damaging chemotherapy because of upregulated BARD1 and BRCA1
Source: Nat Commun. 2018 Apr 23;9:1595. doi: 10.1038/s41467-018-03951-0 (PMC5913295; doi:10.1038/s41467-018-03951-0)
Supplement: Supplementary file 1 — Supplementary Information [file 41467_2018_3951_MOESM1_ESM.pdf]

SUPPLEMENTARY INFORMATION

Tamoxifen-resistant Breast Cancer Cells Are Resistant to DNA-damaging Chemotherapy Because of Upregulated BARD1 and BRCA1

Zhu et al.

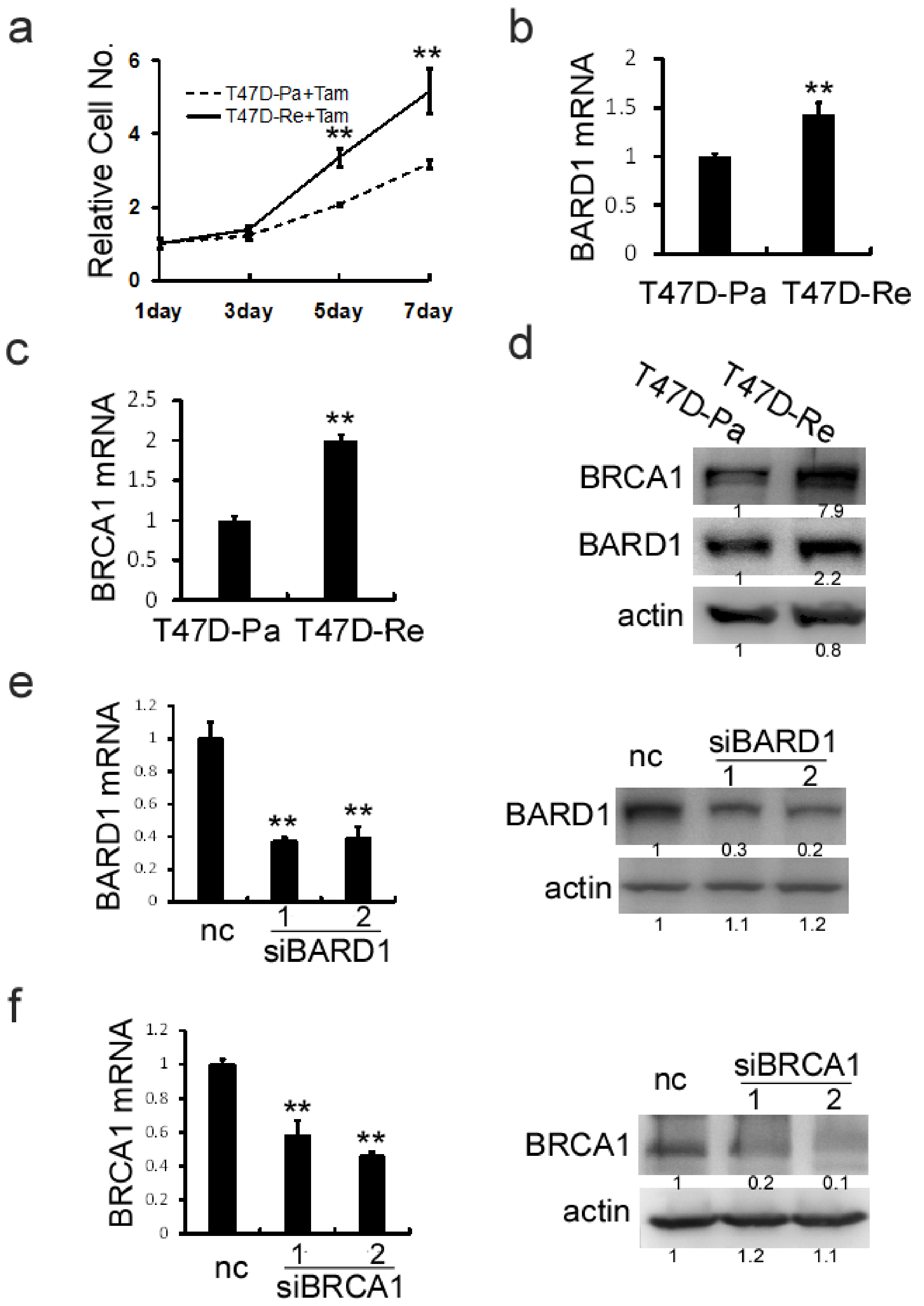

Supplementary Figure 1. BARD1/BRCA1 is upregulated in T47D tamoxifen-resistant breast cancer cells.

(a) The proliferation of T47D-Pa and T47D-Re cells under tamoxifen treatment. **, $p<0.01$; compared with MCF7-Pa by Student's *t* test. (b-d) The mRNA (b,c) and protein (d) expression of BARD1 and BRA1 in T47D-Pa and T47D-Re cells. **, $p<0.01$; compared with MCF7-Pa by Student's t test. (e,f) The mRNA and protein expression of BARD1 (e) and BRCA1 (f) in MCF7-Re cells transfected with negative control siRNA (nc), BARD1 or BRCA1 siRNAs. **, $P<0.01$ compared with nc by Student's *t* test. In a-c, e, f, data show means±s.d. (n=3).

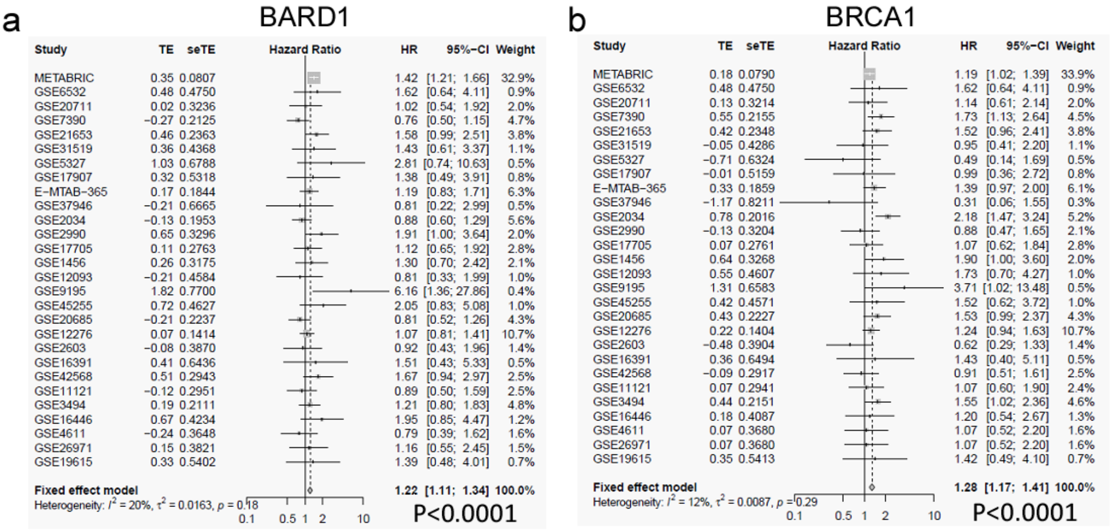

Supplementary Figure 2. Pooled analysis of the association between the expression of BARD1 or BRCA1 and overall survival in 27 breast cancer datasets.

Pooled analysis of the association between expression of BARD1 (a) or BRCA1 (b) and overall survival of breast cancer patients in 27 datasets.

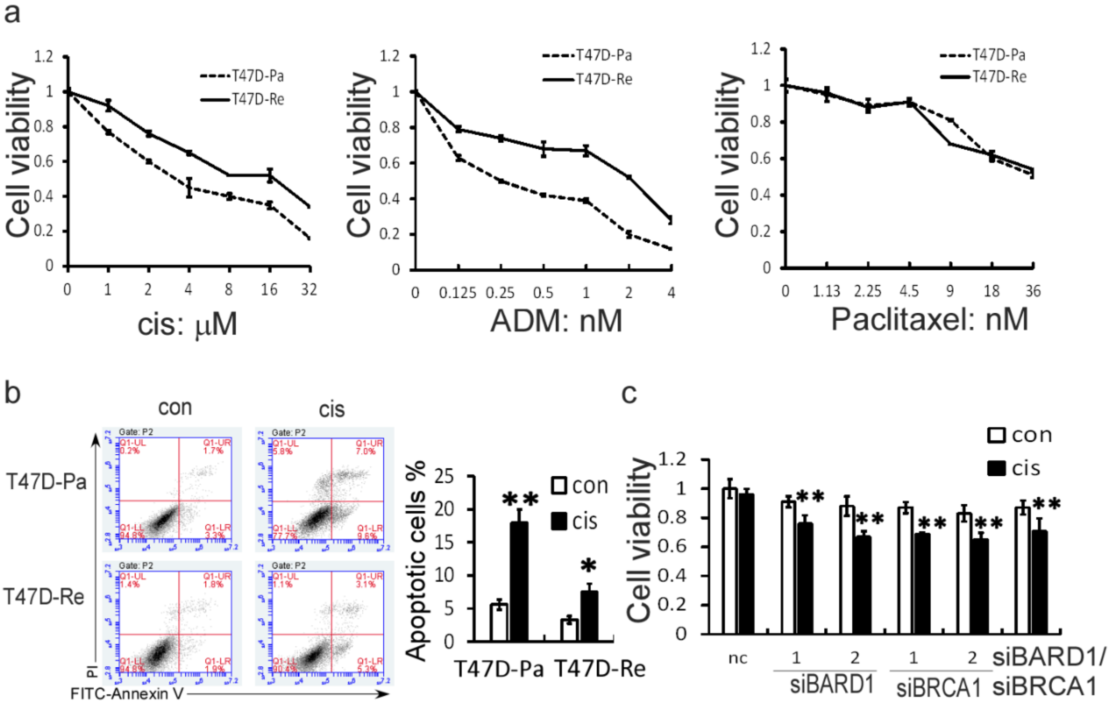

Supplementary Figure 3. T47D tamoxifen resistant cells are resistant to DNA-damaging chemotherapy.

(a) T47D-Pa and T47D-Re cells were treated with different concentrations of cisplatin (cis)、adriamycin (ADM) or paclitaxel, and the proportions of viable cells were examined by cell viability assay. (b) Apoptosis assay by AnnexinV-FITC/PI staining showing the percentages of apoptotic cells when T47D-Pa and T47D-Re cells were treated with cisplatin. (c) Cell viability assay showing the sensitivity to cisplatin in T47D-Re cells when transfected with negative control siRNA (nc), BARD1 or BRCA1 siRNA. In a-c, data show means±s.d. (n=3). **, P<0.01 compared with control by Student's t test.

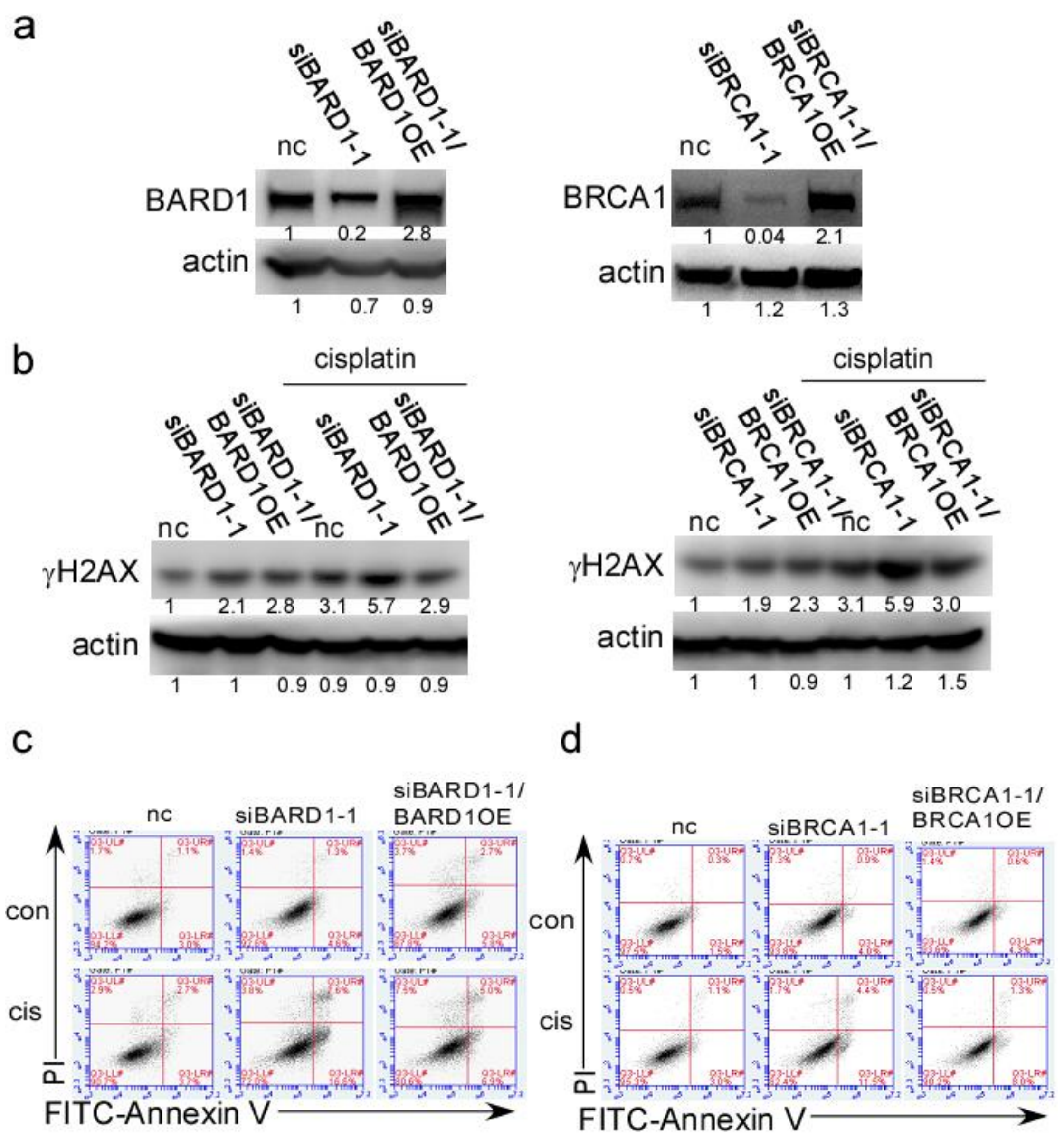

Supplementary Figure 4. The chemoresistance of cisplatin are restored by BARD1 or BRCA1 over expression after BARD1 or BRCA1 knockdown.

(a) Western blot showing the restored expression of BARD1 and BRCA1 in MCF7-Re cells transfected with negative control siRNA, BARD1 or BRCA1 siRNA (targets to 3'-UTR of BARD1 or BRCA1), BARD1 or BRCA1 siRNA combined with pcDNA6b-BARD1 or BRCA1 expression vector. (b-d) The expression of γH2AX (b) and the apoptotic cells (c,d) in MCF7-Re cells transfected with negative control siRNA, BARD1/BRCA1 siRNA or combined with pcDNA6b-BARD1 or BRCA1 expression vector.

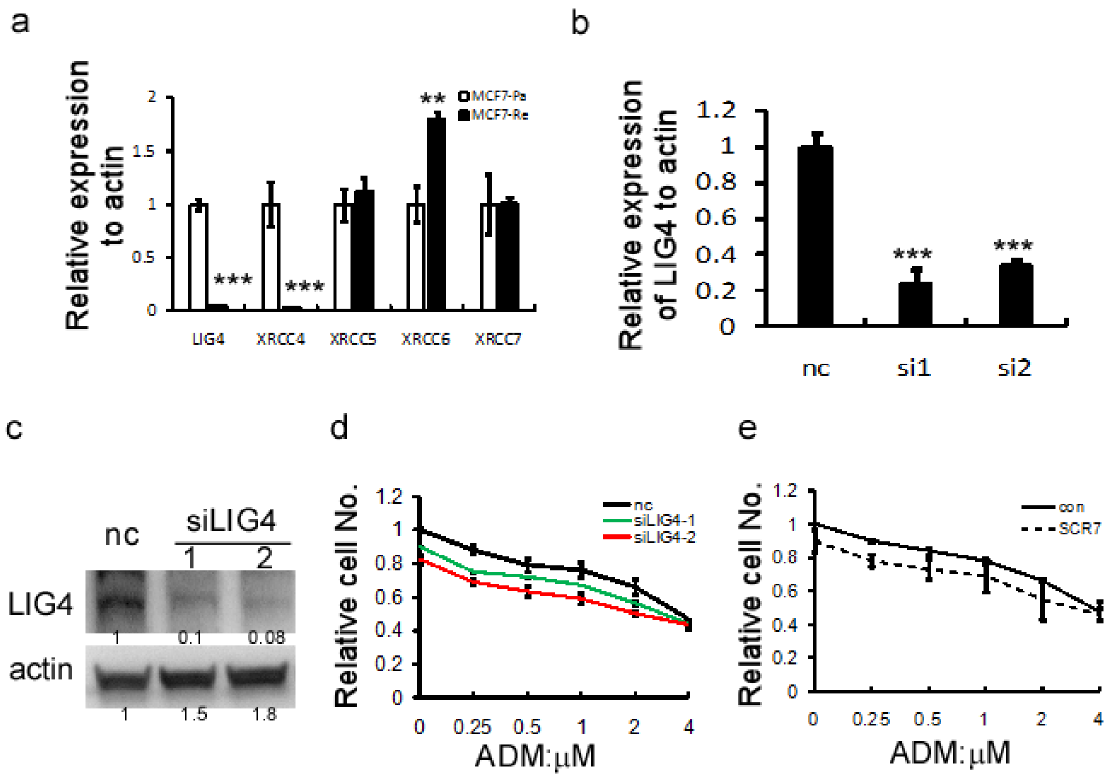

Supplementary Figure 5. NHEJ pathway does not play an important role in chemoresistance of tamoxifen resistant breast cancer cells.

(a) The mRNA expression of NHEJ pathway associated genes including LIG4, XRCC4, XRCC5, XRCC6 and XRCC7 in MCF7-Pa and MCF7-Re cells. ***, $P<0.001$ compared with that in MCF-Pa by Student's *t* test. (b,c) The mRNA (b) and protein (c) expression of LIG4 in MCF7-Re cells transfected with negative control or LIG4 siRNAs. ***, P<0.001 compared with the nc by Student's *t* test. (d,e) Cell viability assay showing the sensitivity to adriamycin in MCF7-Re cells when transfected with negative control siRNA, LIG4 siRNAs (d) or treated with LIG4 inhibitor SCR7 (e). In a, b, d, e, data show means±s.d. (n=3).

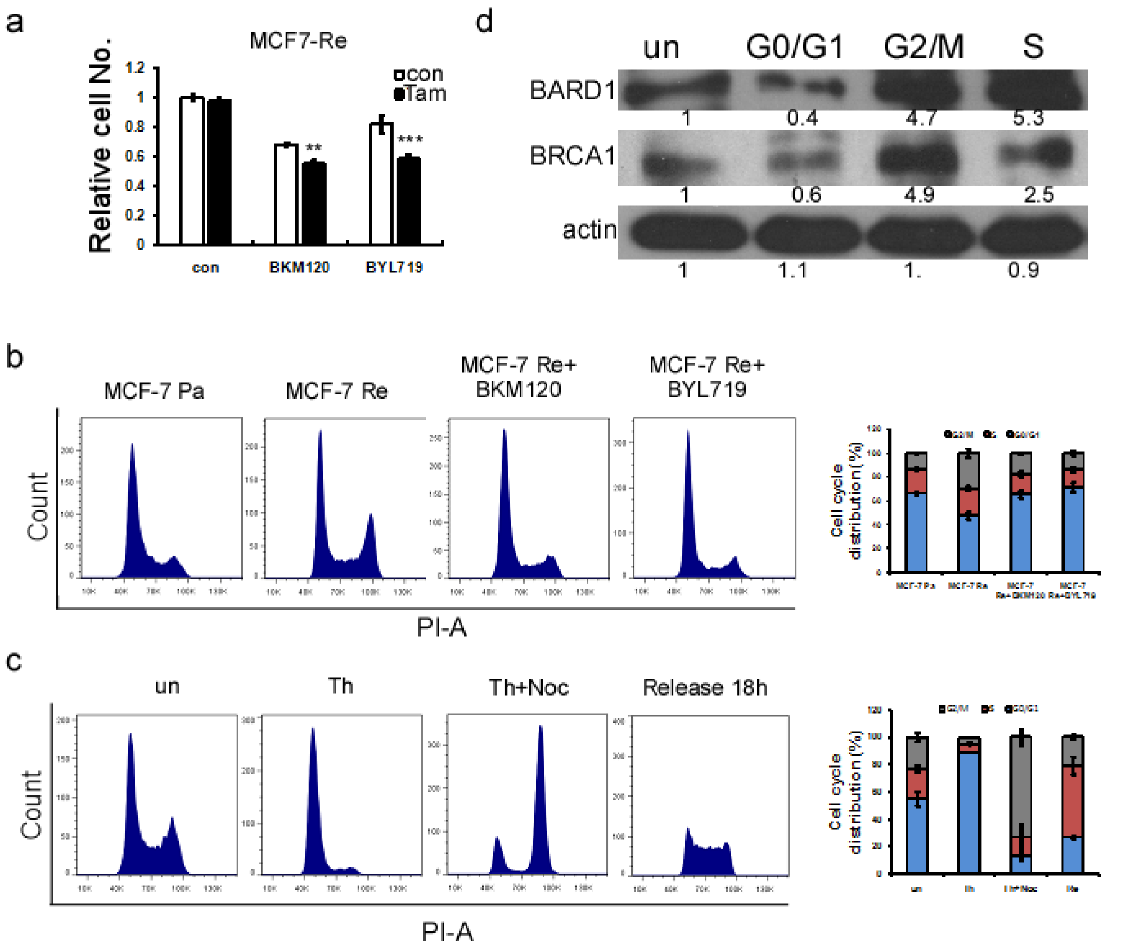

Supplementary Figure 6. BARD1 and BRCA1 are high expressed in G2/M and S phase.

(a) Cell viability assay showing the sensitivity to tamoxifen in MCF7-Re cells treated with control (con), BKM120, BYL719. ***P*<0.01, ***, P<0.001 compared with that in con by Student's *t* test. (b) Cell cycle distribution in MCF7-Pa, MCF7-Re cells, or MCF7-Re cells treated with BKM120 or BYL719. (c)The cell cycle distribution of MCF7-Re cells after using thymidine (Th) and/or nocodazole (NOC) to enrich cells in G0/G1, G2/M and S phases. (d) The expression of BARD1, BRCA1, and GAPDH in MCF7-Re cells unenriched or enriched in G0/G1, G2/M and S phases. In a, b,c, data show means±s.d. (*n*=3).

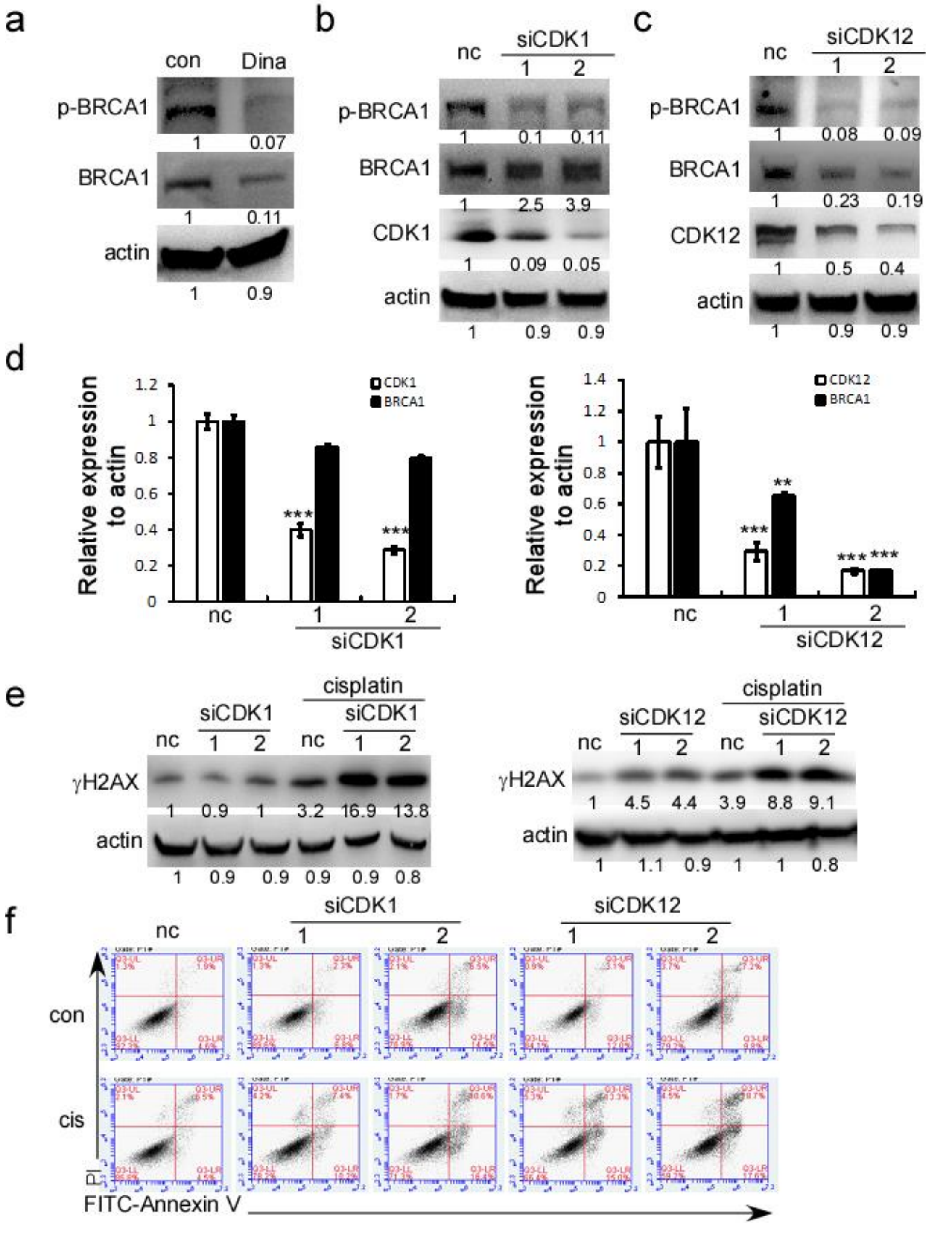

Supplementary Figure 7. Dinaciclib targets both CDK1 and CDK12 to regulate BRCA1 expression. (a) The expression of total and phosphorylated BRCA1 (p-BRCA1) in MCF7-Re cells treated with control (con) or Dinaciclib (Dina). (b,c) The expression of total BRCA1 and p-BRCA1 in MCF7-Re cells transfected with negative control siRNA (nc), siRNAs targeting CDK1 or CDK12. (d) The mRNA expression of BRCA1 and CDK1 or CDK12 in MCF7-Re cells transfected with negative control siRNA, siRNAs targeting CDK1 or CDK12. (e,f) The expression of γH2AX (e) and the apoptotic cells (f) induced by cisplatin when MCF7-Re cells were transfected with negative control siRNA, siRNAs targeting CDK1 or CDK12. In d, data show means±s.d. (*n*=3). **, *P*<0.01; ***, P<0.001 compared with nc by Student's *t* test.

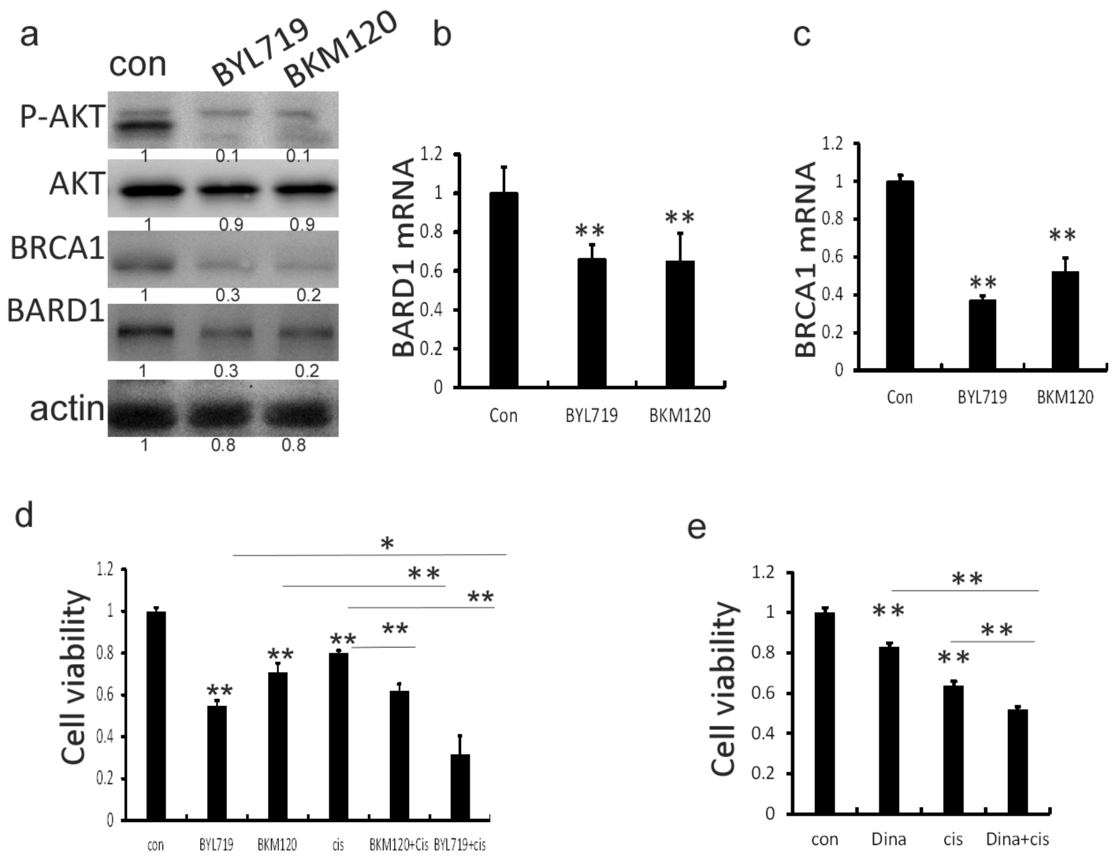

Supplementary Figure 8. PI3K inhibitor restores the sensitivity to cisplatin in T47D tamoxifen resistant cells.

(a) Western blot showing the expression of BARD1, BRCA1, total and phosphorylated AKT in T47D-Re cells treated with control, BKM120 or BYL719. (b,c) qPCR showed the mRNA level of BARD1 (b) and BRCA1 (c) in T47D-Re cells treated with BKM120 or BYL719. (d) Cell viability assay showing the sensitivity to cisplatin in T47D-Re cells treated with control, BKM120 or BYL719. (e) Cell viability assay showing the sensitivity to cisplatin in T47D-Re cells treated with control or Dinaciclib (Dina). In b-e, data show means±s.d. (n=3). *, P<0.05; **, P<0.01 compared with control or indicated lines by Student's t test.

Figure 1d

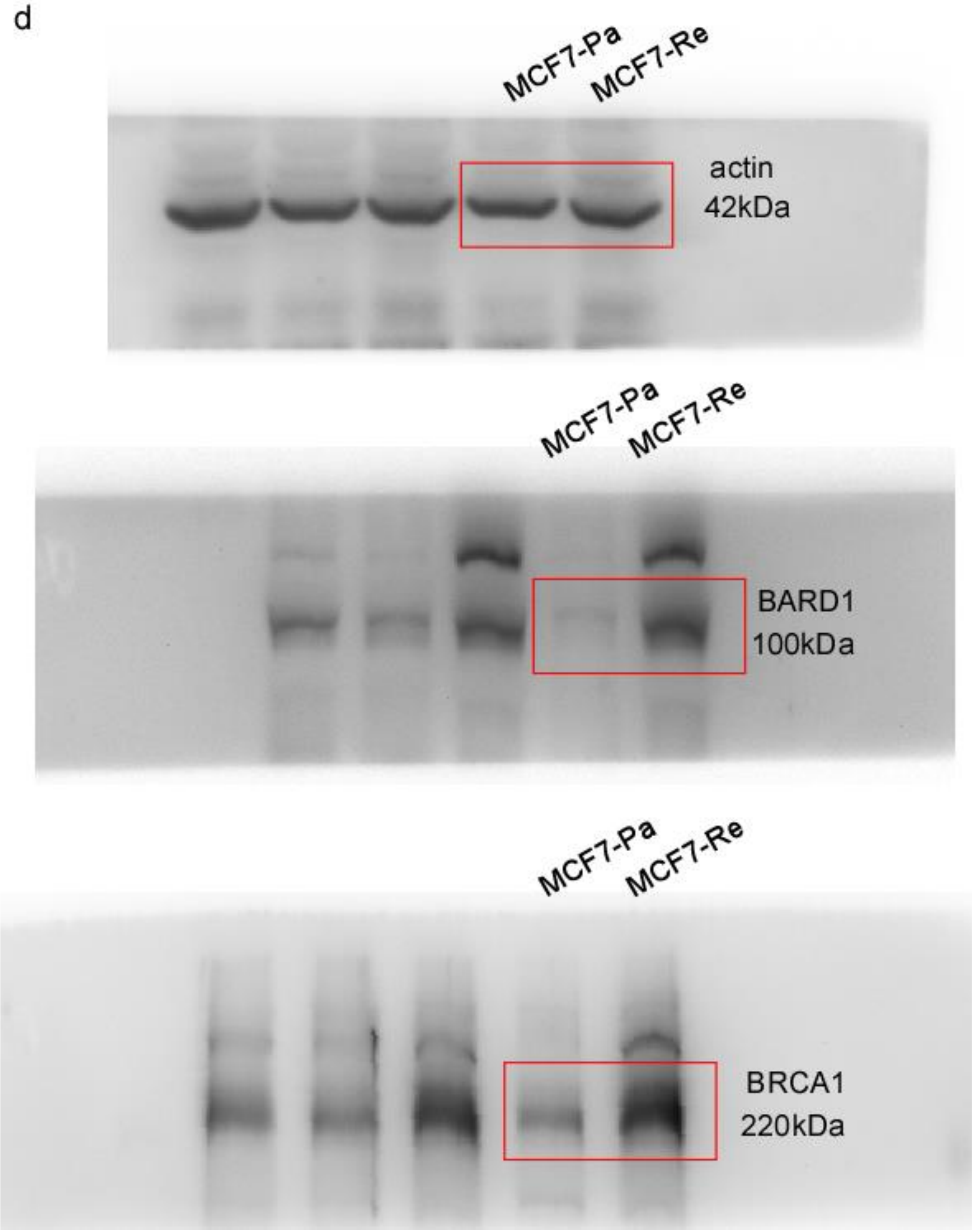

Supplementary Figure 9. Original uncropped gels for Fig. 1d. Original gels for the western blots present in Fig. 1. Area cropped in indicated with red box, individual molecular weights of each individual antibody indicated on western blot.

Figure 2e

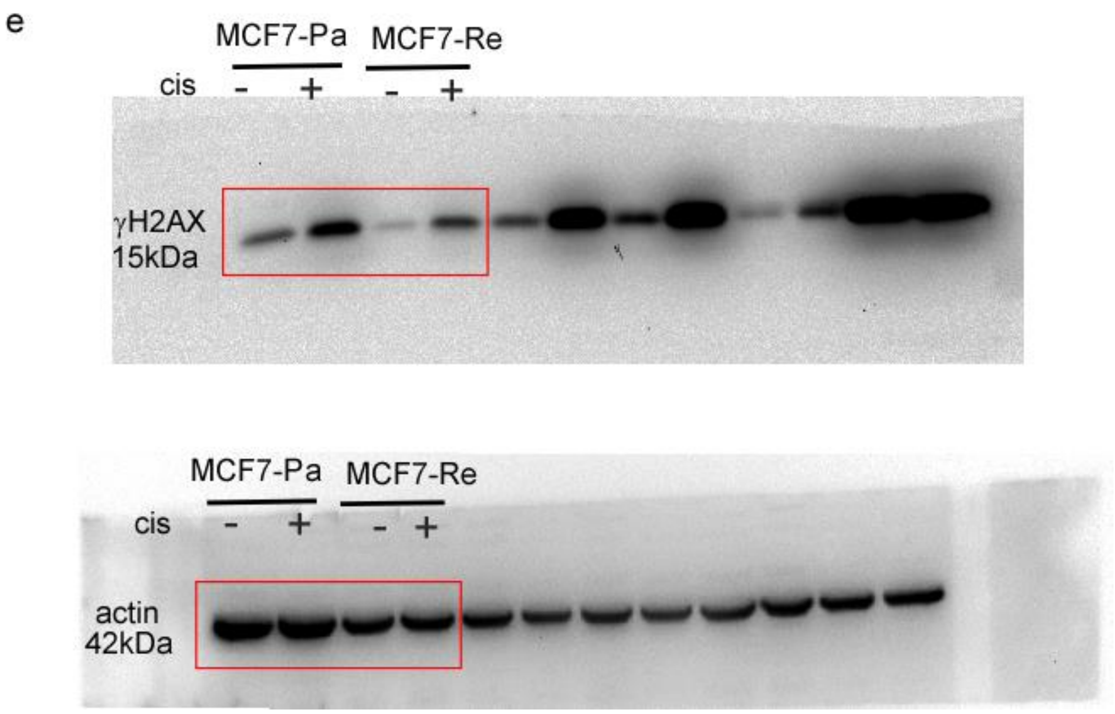

Supplementary Figure 10. Original uncropped gels for Fig. 2e. Original gels for the western blots present in Fig. 2. Area cropped in indicated with red box, individual molecular weights of each individual antibody indicated on western blot.

Figure 3d

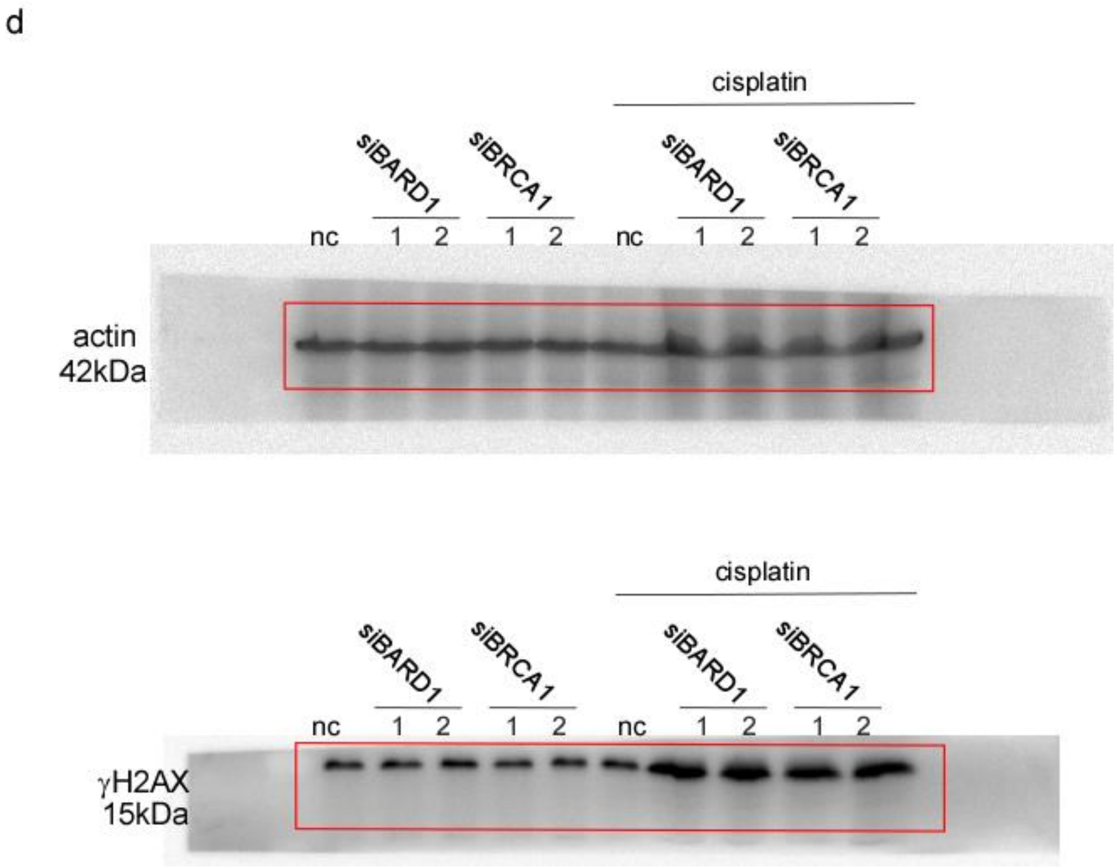

Supplementary Figure 11. Original uncropped gels for Fig. 3d. Original gels for the western blots present in Fig. 3. Area cropped in indicated with red box, individual molecular weights of each individual antibody indicated on western blot.

Figure 4a

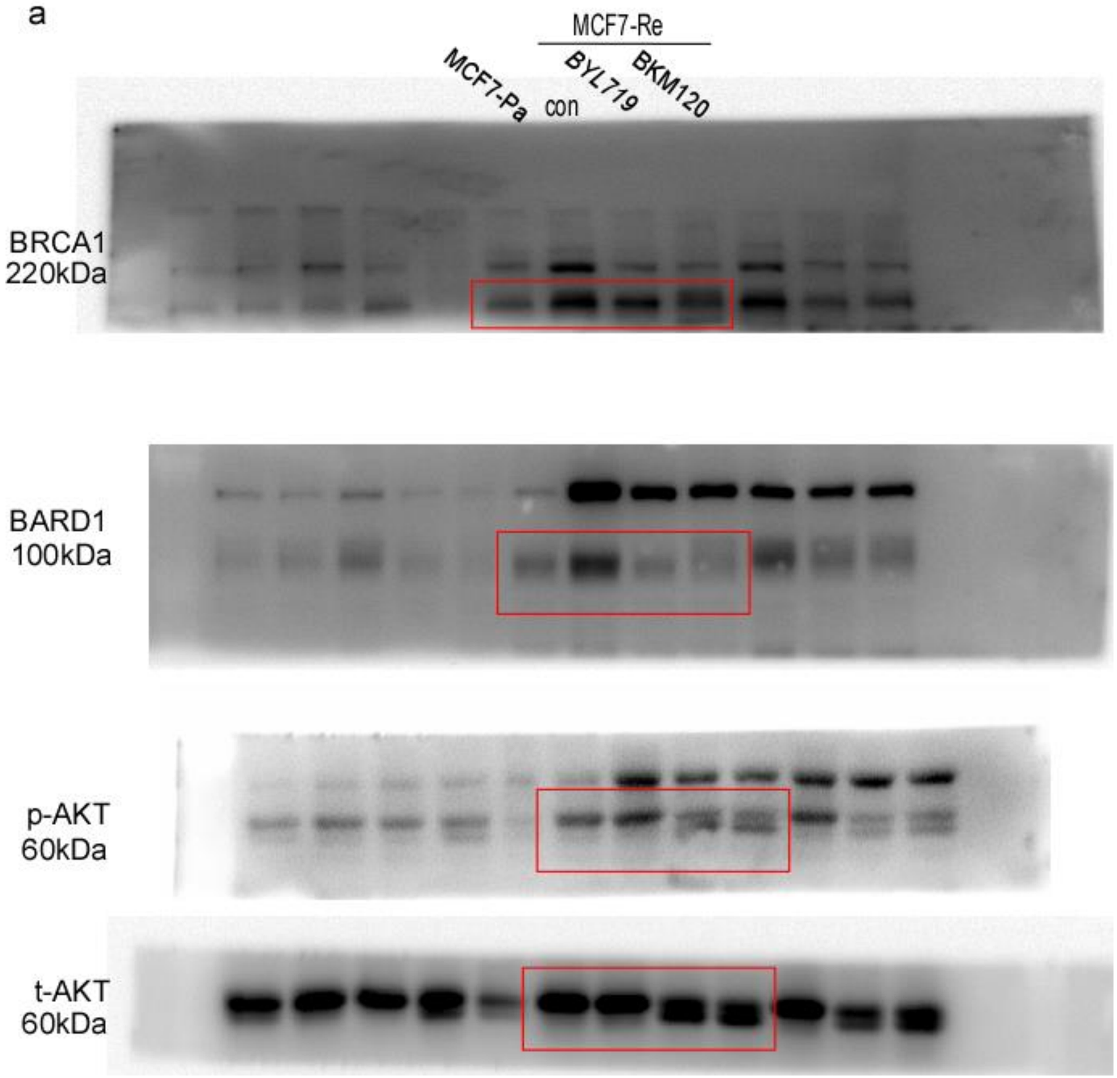

Supplementary Figure 12. Original uncropped gels for Fig. 4a. Original gels for the western blots present in Fig. 4. Area cropped in indicated with red box, individual molecular weights of each individual antibody indicated on western blot.

Figure 4e and Figure 4i

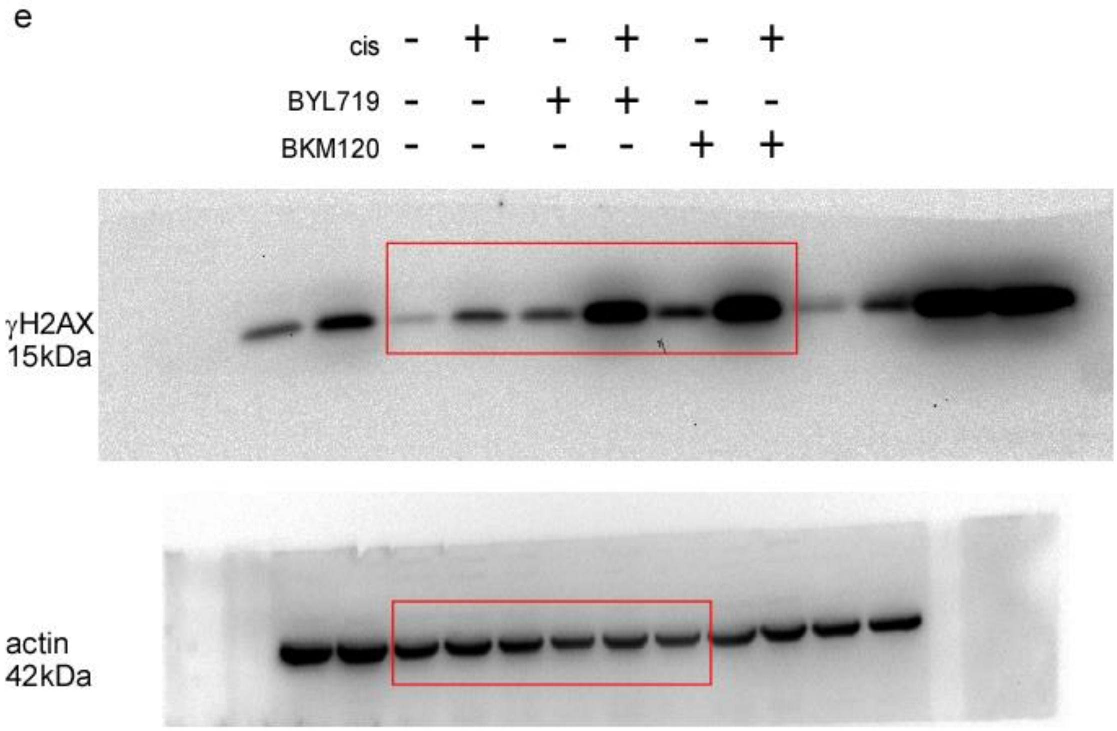

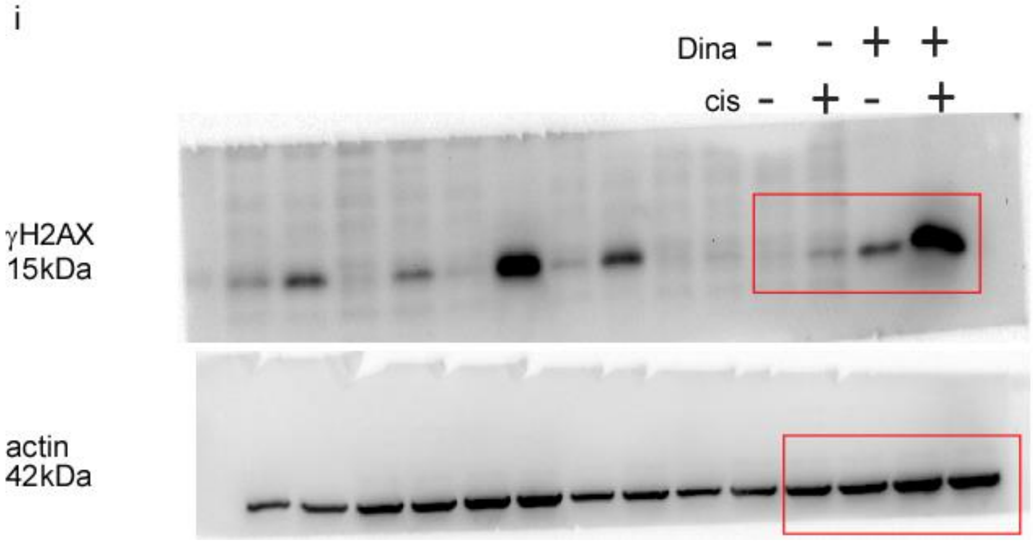

Supplementary Figure 13. Original uncropped gels for Fig. 4e and Fig. 4i . Original gels for the western blots present in Fig. 4. Area cropped in indicated with red box, individual molecular weights of each individual antibody indicated on western blot.
